# A Study of the Management of Patients Taking Novel Oral Antiplatelet or Direct Oral Anticoagulant Medication Undergoing Dental Surgery in a Rural Setting

**DOI:** 10.3390/dj3040102

**Published:** 2015-10-06

**Authors:** Steven Johnston

**Affiliations:** Balfour Oral Surgery Unit, NHS Orkney, Balfour Hospital, Kirkwall, Orkney KW15 1BH, Scotland, UK; E-Mail: steven.johnston@nhs.net; Tel.: +44-18-5688-8258

**Keywords:** NOAP, DOAC, Target-specific Oral Anticoagulants (TSOAC), Novel Oral Anticoagulants (NOAC), prasugrel, ticagrelor, dabigatran, rivaroxaban, apixaban

## Abstract

Purpose: Novel oral antiplatelet (NOAP) (prasugrel and ticagrelor) and direct oral anticoagulant drugs (DOAC) (dabigatran, rivaroxaban and apixaban) have emerged in the last decade. This study was undertaken to determine current approaches taken to the management of patients taking these agents in dental practice in a remote and rural setting. Methods: A small retrospective study was carried out in a small island population that identified patients taking one of the above drugs. All national health service and private dental records were examined to determine the type of treatment carried out and whether drug therapy, treatment plans or actual treatment were modified as a result of NOAP or DOAC therapy. In addition other outcomes such as referral to another service for advice or treatment and any adverse bleeding events were noted. Results: 156 dental encounters for 95 patients taking one of the drugs were identified. Significant events were identified in sixteen encounters and the management of patients taking each drug type differed significantly between cases but no patients returned with troublesome post-operative bleeding. Conclusions: The approaches taken by dental surgeons in Orkney in the management of the NOAPs and DOACs varied and this is likely to be a reflection of the limited literature available.

## 1. Introduction

Over the past decade a number of new drugs have emerged which pose a risk to patients undergoing invasive dental procedures. Due to limitations with traditional mono- and dual antiplatelet therapies such as with aspirin and clopidogrel, two new agents have been developed [1]. The novel oral antiplatelets (NOAP) prasugrel (Effient^®^, Lilly USA, LLC, Indianapolis, IN, USA) and ticagrelor (Brilique™, AstraZeneca, London, United Kingdom) are claimed to have superior clinical properties to traditional agents [2] and are increasing in popularity, with the National Institute for Health and Care Excellence (NICE) approving either for use in combination with aspirin for a subset of acute coronary syndromes [3,4]. Warfarin, which has been the mainstay of anticoagulation therapy for decades, is being replaced with newer agents which differ in that they target a single factor within the coagulation cascade directly and reach a more predicable peak plasma concentration so can be taken at a fixed daily dose with no need for blood monitoring [5,6,7]. These drugs have been termed direct, target-specific or novel oral anticoagulants (DOAC, TSOAC or NOAC respectively) but the International Society on Thrombosis and Haemostasis Scientific and Standardisation Committee have settled on DOAC as the preferred term [8]. The three drugs currently available are dabigatran (Pradaxa^®^, Boehringer Ingelheim, Ingelheim, Germany) (a direct thrombin inhibitor), rivaroxaban (Xarelto®, Bayer, Leverkusen, Germany) and apixaban (Eliquis®, Bristol-Myers Squibb, Uxbridge, United Kingdom) (both factor Xa inhibitors, FXaIs). The main disadvantages of these drugs are the lack of a reversal agent (although agents are currently under development [9,10,11]) and the difficulties in assessing the level of anticoagulation as traditional tests such as INR are not reliable [12,13,14,15]. Prothrombin time (PT) is not sensitive to dabigatran induced anticoagulation but activated partial throboplastin time (aPTT) may be used with caution in establishing over-coagulation in emergency situations or in centres where other tests are not available. Thrombin (clotting) time (TT/TCT) and ecarin clotting time (ECT) are more sensitive methods of testing anticoagulation in DTIs but are not widely available [12,13]. Apixaban can prolong PT (depending on the laboratory reagent used) and increase aPTT but does so to varying degrees. Rivaroxaban has a dose dependent effect with PT with most laboratory reagents. However, to achieve an accurate assessment of anticoagulant effect, chromogenic anti Xa assays, which are not universally available, are required [13,14,15]. Another disadvantage of dabigatran is that 80% of the drug is cleared via the kidneys and therefore caution is required in patients with renal failure [16]. The FXaIs can be used with caution (possibly at a reduced dose) in mild or moderate hepatic or renal failure but should be avoided in severe renal failure [6,7,13]. The DOACs have been approved by NICE in the management of patients with atrial fibrillation and in the prevention and management of venous thromboembolism in patients undergoing orthopaedic surgery [17,18,19,20,21,22,23,24], although the indications are expanding. Following a number of clinical trials [25,26], another FXaI edoxaban (Savaysa [US] or Lixiana [EU]) produced by Daiichi Sankyo has recently been approved by the FDA, has been granted approval marketing authorisation in Europe and is currently being considered by NICE for use in the management of patients with AF and in the treatment of VTE [27,28,29]. Betrixaban (proprietary name to be confirmed) from Portola Pharmaceuticals, has completed phase-2 trials for prevention of VTE in AF patients [30] and for the prevention of VTE following orthopaedic surgery [31] and is entering phase 3 trials for its use in the long-term prevention of VTE in medically ill patients [32].

The management of patients taking a NOAP or DOAC is not clear in the literature and traditional approaches, such as those taken with older agents may no longer be appropriate. This study aims to establish the current approaches when encountering patients taking these drugs in a rural setting.

## 2. Materials and Methods

A simple observational study of patients taking a NOAP or DOAC was selected as the most straight-forward method to achieving the objective. The first task was to identify all patients in Orkney taking the NOAPs prasugrel and ticagrelor and the DOACs dabigatran, rivaroxaban and apixaban. A list of unique patient identifiers (Community Health Index (CHI) numbers) for all patients in Orkney taking any of the five drugs during April-September 2014 was obtained from the National Health Service (NHS) Orkney central pharmacy using data from Information Services Division (ISD), NHS Scotland. The disadvantage of using this source was that the data were approximately five months out of date, so any patient who had commenced the drug after this time and had a dental appointment would be missed. After the target group were identified, access was required to the dental records held at all three independent dental practices in Orkney (with permission from the practice owners) and those of the NHS Orkney Public Dental Service (PDS), encompassing all private and dental services provided in Orkney.

The medical history section of each record indicated the first attendance following commencement of the drug and all appointments from this date onwards until the March 2015 were studied, unless there was an entry to suggest the drug had been discontinued. Patients who had not seen a dentist since commencement of the drug were excluded from the study. Each record was studied to identify what dental treatment (invasive or otherwise) the patient had whilst taking the drug and whether any adverse bleeding effects were noted. In addition, the following specific criteria were recorded:

- Modification of the normal drug dose or a period of discontinuation (and if so, details provided);
- Modification of the treatment plan (*i.e.*, avoidance of invasive options);
- Modification of the treatment carried out (*i.e.*, additional measures to ensure haemostasis);
- Referral to another service;
- Seeking advice from another clinician/specialist.

The information was collected on a spreadsheet (Microsoft^®^ Office Excel 2003, Redmond, WA, USA) and once all of the data was collected and collated, the CHI numbers were removed and the subjects were allocated a three digit code. The first digit of the code indicated the drug each subject was taking (1 = prasugrel, 2 = ticagrelor, 3 = dabigatran, 4 = rivaroxaban and 5 = apixaban) and the remaining two digits ascended from 01 upwards in no particular order.

A full application was submitted to the NHS Research Ethics Committee (REC) to obtain ethical approval and permission to embark on the project. The application was assessed prior to the scheduled REC meeting and permission to proceed was granted without the need for full REC assessment, subject to Caldicott Guardian approval, and without the need for patient consent.

## 3. Results

From a population of approximately 21,000, 95 patients were identified from the pharmacy data. No patients in Orkney were found to be taking prasugrel and 32 patients were taking ticagrelor. Of the DOACs dabigatran, rivaroxaban and apixaban, 13, 43 and 7 patients were taking these drugs respectively. No patients were found to be taking a combination of DOACs and NOAPs. A total of 51 patients had not been seen by a dentist or dental care professional since starting the drug and were excluded from the study. The remaining 44 patients attended a total of 156 dental appointments with the number of visits per patient ranging from 1 to 12 and an age range of 40–90, with a mean age of 64 years old. No dental encounters were found for patients taking apixaban. Table 1 shows the proportion of each drug within the 156 appointments studied.

**Table 1:** Distribution of patients taking a Novel oral antiplatelet (NOAP) or direct oral anticoagulant drugs (DOAC) in Orkney who attended a dental practice.

Drug	Number (*n*)	Percentage of Appointments (%)
**Prasugrel**	0	0
**Ticagrelor**	55	35
**Dabigatran**	32	21
**Rivaroxaban**	69	44
**Apixaban**	0	0

Only 13 (8.3%) of the encounters were invasive with the majority (eleven) being dental extractions. The remaining two invasive procedures were an apicectomy of an upper incisor and an incisional biopsy of the tongue, both in patients taking rivaroxaban. Adrenaline-containing local anaesthetic with a concentration ranging between 1:80,000 and 1:200,000 was used in all invasive procedures.

One or more of the criteria were present in 12 cases and overall there were 16 appointments with significant events or findings, involving a total of 11 patients, as summarised in Table 2 below. Worryingly, five invasive procedures were carried out with no mention of the drug in the clinical notes (although it was recorded in the medical history section) and no additional measures to reduce bleeding risk were planned prior to the procedure. This suggested the drug had not been identified as an antiplatelet or anticoagulant agent. Adverse peri-operative bleeding was observed in two of these cases: one patient (taking dabigatran) who had two extractions on separate visits. In both instances excessive immediate post-extraction bleeding was noted and then successfully controlled following the placement of haemostatic sponge. None of the patients in this study returned, either to their own GDP, the oral surgery department or to the on-call/out of hours dental service, with bleeding issues.

**Table 2:** Notable interventions and specific treatment details.

Drug	Patient ID (Visit No.)	Details
**Ticagrelor**	201 (6)	Extraction with Gelatemp (Coltene-Whaledent^®^, Cuyahoga Fall, OH, USA) haemostatic spongeplacement.
	201 (9)	Extraction with no (additional local haemostatic measures (ALHMs). General dental practitioner (GDP) remarked that there was no adverse peri-operative bleeding so opted against additional measures.
	208 (1)	Patient presented in pain but GDP unsure about safety of extraction so contacted the patient’s general medical practitioner (GP) and cardiologist and the local oral surgery department for advice: postponed extraction, gave antimicrobials and arranged review.
	208 (2)	On review the pain had subsided and following the advice from the above clinicians, extraction was postponed until patient was deemed more stable (recent myocardial infarction).
	220 (2)	Extraction performed with no change to patient management.Drug was recorded in the medical history section but it was not mentioned in the dental case notes.
	227 (1)	Seen by GDP and agreed extraction necessary. GDP did not wish to proceed due to uncertainty about peri-operative bleeding and referred the patient to the local oral surgery department.
	227 (2)	The oral surgery department then extracted the tooth, placed Surgicel^®^ (Ethicon US, LLC, Boston, MA, USA) and sutured the socket.
**Dabigatran**	303 (5)	Extraction performed with haemostatic sponge placement (type not specified). The drug was stopped for 48hours prior to procedure
	304 (7)	Extraction performed with no additional haemostatic measures.The drug was stopped for 48hours prior to the procedure and it was noted that the patient had a history of impaired renal function. The drug was recommenced that evening.
	305 (1)	Extraction performed and increased immediate post-operative bleeding noted which responded to placement of Gelatemp (Coltene-Whaledent^®^) haemostatic sponge. Drug was recorded in the medical history section but not mentioned in the dental case notes
	305 (2)	Extraction performed and once again increased immediate post-operative bleeding noted which responded to placement of Gelatemp (Coltene-Whaledent^®^) haemostatic sponge. The drug was recorded in the medical history section but it was not mentioned in the dental case notes.
**Rivaroxaban**	404 (2)	Extraction carried out and no additional haemostatic measures used. The GDP remarked that the tooth had severe periodontal disease and the socket had almost entirely epithelialised resulting in little bleeding. The drug was stopped 24 hours prior to procedure (on the GDPs advice) and recommenced shortly after.
	407 (7)	Incisional biopsy lateral border of tongue. Sutures used but no additional haemostatic measures. No mention of drug in the case notes and the drug was not discontinued.
	422 (2)	Periradicular surgery on an upper incisor performed under general anaesthetic using a 2-sided mucoperiosteal flap with no additional haemostatic measures required.The drug was stopped 48hours prior to the procedure on the advice of an anaesthetist.
	430 (6)	Extraction performed with no reference to the drug in the notes and no additional haemostatic measures.
	432 (2)	The patient required multiple extractions but the GDP opted to perform a single “test” extraction first. No additional haemostatic measures were used.

## 4. Discussion

### 4.1. Study Limitations

The study had a number of limitations, with the most striking being the low number of subjects. Orkney has a population of approximately 21,000 and only 95 patients taking either a NOAP or DOAC were identified. The study may, however, serve as a pilot for similar methods to be used on a larger population in the future, possibly with a warfarin control group. If the study was conducted again after several years, the number of dental interventions would be greater and the number of patients taking a NOAP or DOAC (possibly including newer agents not included in this study) would be higher.

Interestingly, one of the small island general practitioners (GPs) had no patients taking any of the NOAPs or DOACs due to concerns about the drugs in combination with delays in access to hospital care for geographical reasons. Orkney has a small rural general hospital with out of hours GP and dental services, an on-call surgeon and an on-call hospital physician and many emergencies can be dealt with on-site. However, occasionally, for some specialist interventions, including certain emergency situations, patients have to travel up to 140 miles by road and air to Aberdeen Royal Infirmary, or further if a resident of an island not connected to the Orkney mainland, which could take several hours. Therefore the risks that may be accepted in more urban populations may not be reasonable in a rural area and it may not be appropriate to generalise the findings of this island population study.

Another potential issue with this study was the assumption that the medical history was kept up to date, allowing identification of when the patient first had a dental intervention whilst taking the NOAP or DOAC. The patient may have received dental treatment prior to the date the drug was recorded and therefore the study may have missed interventions. Conversely, the patient may have stopped the drug and the study incorrectly recorded it as an intervention.

The final issue was patient follow-up. Accident and emergency or hospital records were not accessed to capture those patients who may have had bleeding addressed without dental input or those patients who had thromboembolic complications. Patient feedback was not sought to assess whether bleeding was encountered which did not necessitate any medical or dental intervention. Therefore it is feasible that patients may have had bleeding issues which were not captured.

### 4.2. Orkney Study *versus* Current Evidence

The approaches to managing dental patients taking a NOAP or a DOAC in the Orkney study were variable and this is likely to be a reflection of the differing (and often conflicting) guidance within the limited scientific literature available. It would be hoped that following publication of national guidance [33], awareness of the drugs will increase and the management of these patients will become standardised. It will then be beneficial, preferably using large population studies, to quantify the risk of both bleeding and thromboembolic events to assess the safety of the approach. However, even in the absence of clinical guidelines, no significant peri-operative bleeding was reported in Orkney and no thromboembolic events were known to have occurred. The latter must clearly be treated with caution due to the low numbers and study limitations but it is still an encouraging finding.

## 5. Conclusions

The varied approaches to dental surgery in patients on NOAP or DOAC drugs in this small study of a rural area demonstrates that the dental community is in need of guidance and standardised protocols would be welcomed. The lack of scientific literature available is concerning and dental research in this area would be useful but reassuringly, in the small number of cases seen in this study, no adverse post-operative bleeding was recorded.
